# Holistic burden of illness in patients with endogenous Cushing's syndrome: A systematic literature review

**DOI:** 10.1002/edm2.464

**Published:** 2023-12-20

**Authors:** Gabrielle Page‐Wilson, Bhagyashree Oak, Abigail Silber, Janetricks C. Okeyo, Nancy Ortiz, Matthew O'Hara, Stephen Moloney, Eliza B. Geer

**Affiliations:** ^1^ Division of Endocrinology Columbia University Irving Medical Center New York New York USA; ^2^ Trinity Life Sciences Waltham Massachusetts USA; ^3^ Formerly at Strongbridge Biopharma plc, a wholly owned subsidiary of Xeris Biopharma Holdings, Inc. Trevose Pennsylvania USA; ^4^ Multidisciplinary Pituitary and Skull Base Tumor Center Memorial Sloan Kettering Cancer Center New York New York USA

**Keywords:** burden of illness, Cushing's syndrome, patient reported outcome measures

## Abstract

**Objective:**

The objective of this systematic literature review (SLR) was to summarize the latest studies evaluating the burden of illness in endogenous Cushing's syndrome (CS), including the impact of CS on overall and domain‐specific health‐related quality of life (HRQoL) and the economic burden of CS to provide a holistic understanding of disease and treatment burden.

**Methods:**

An SLR was conducted in PubMed, MEDLINE and Embase using the Preferred Reporting Items for Systematic Reviews and Meta‐Analyses (PRISMA) checklist to identify peer‐reviewed manuscripts and conference abstracts published in English from 2015 to December 4, 2020.

**Results:**

Forty‐five publications were eligible for inclusion; data were extracted from 37 primary studies while 8 SLRs were included for reference only. Thirty‐one studies reported HRQoL using validated patient reported outcome (PRO) measures in pre‐ or post‐surgery, radiotherapy and pharmacotherapy patients. Overall, this SLR found that patients with CS have worse outcomes relative to healthy populations across specific dimensions, such as depression, despite an improvement in HRQoL post‐treatment. These findings reveal that CS symptoms are not fully resolved by the existing care paradigm. Few studies report on the economic burden of CS and currently available data indicate a high direct healthcare system cost burden.

**Conclusions:**

Patients with CS experience a significant, complex and multifactorial HRQoL burden. Symptom‐specific burden studies are sparse in the literature and the understanding of long‐term CS symptomatic burden and economic burden is limited. This review intends to provide an updated reference for clinicians, payers and other stakeholders on the burden of CS as reported in published literature and to encourage further research in this area.

## Graphical abstract

Patients with Cushing's syndrome (CS) experience a significant, complex and multifactorial health related quality of life burden. Symptom‐specific burden studies are sparse in the literature and our understanding of long‐term CS symptomatic burden and economic burden is limited. This systematic literature review intends to provide an updated reference for clinicians, payers and other stakeholders on the burden of CS as reported in published literature and to encourage further research in this area.

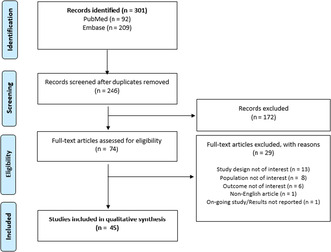

## 1 INTRODUCTION

Endogenous Cushing's syndrome (CS) is a rare condition caused by chronic oversecretion of cortisol. The estimated annual incidence is 1.8–3.2 cases per million,^1^, ^2^ with approximately 70% of cases occurring in females. Based on aetiology, CS can be classified as adrenocorticotropic hormone (ACTH)‐dependent or ACTH‐independent. ACTH‐independent CS is most commonly caused by a cortisol producing adrenal adenoma, and rarely by adrenal cortical carcinoma, or bilateral disease such as macronodular adrenal hyperplasia or primary pigmented nodular adrenocortical disease.^3^ ACTH‐dependent CS accounts for 80% of cases and is caused by either pituitary or ectopic ACTH producing tumours. Cushing's disease (CD), the most common form of endogenous CS, is caused by an ACTH‐secreting pituitary adenoma that stimulates the adrenal overproduction of cortisol.^3^

Chronic hypercortisolism can lead to multisystem morbidities due to the ubiquitous downstream effects of cortisol. Patients with CS present with a variety of clinical manifestations of cortisol excess, including mood, reproductive, skin, neurocognitive and metabolic symptoms, many of which are common in the general population.^4^ Current treatment options for CS include transsphenoidal surgery as first‐line treatment, followed by pharmacotherapy, bilateral adrenalectomy, and pituitary radiotherapy, as second‐line treatment options. Available medical therapies include steroidogenesis inhibitors (e.g. ketoconazole, levoketoconazole, etomidate, metyrapone and osilodrostat), glucocorticoid receptor antagonists (e.g. mifepristone) and tumour directed therapies (e.g. pasireotide and cabergoline). These medications are administered either as mono‐ or combination therapies and may be used to treat CS prior to surgery, when surgery is contraindicated, when remission is not achieved post‐operatively, or in the setting of disease recurrence while awaiting the effects of radiation therapy.^5^ Despite the available treatment options, there remains a clear need to improve the burden of illness in Cushing's syndrome, as it relates to health‐related quality of life (HRQoL) and economic burden of the disease and its treatment.^6^

Previously published systematic literature review (SLR) studies have examined the literature on the burden of illness in patients with CS or CD. However, many SLRs were limited in their approach. Several studies describing patients with CD were actually designed to evaluate the burden of illness among patients with all types of pituitary tumour subtypes while other studies focused solely on specific symptoms instead of the holistic burden.^7^, ^8^ The objective of this SLR is to provide a comprehensive, up‐ to‐date summary of the latest studies evaluating the burden of illness in CS, including the impact of CS on overall HRQoL and on specific domains of health such as anxiety, depression and fatigue. In addition, this study aims to summarize evidence on the economic burden of CS experienced by patients, in terms of the direct (e.g. hospitalization, outpatient visits and pharmacy costs) and indirect costs (e.g. loss of productivity, loss of pay and unpaid work) associated with treatment or management of the condition, with the goal of providing a holistic understanding of both disease and treatment burden and the impact of each on patients.

## 2 MATERIALS AND METHODS

This SLR study was conducted between October 2020 and April 2021 according to the Preferred Reporting Items for Systematic Reviews and Meta‐Analyses (PRISMA) guidelines and conducted by the full team of study authors.^9^ Select findings from this SLR were presented as posters at the European Neuroendocrine Association (ENEA) Workshop in 2021 and the International Society for Pharmacoeconomic and Outcomes Research (ISPOR) Conference in 2022.^10^, ^11^ The SLR protocol was not registered as the review aimed to synthesize qualitative data and a follow‐up meta‐analysis was not planned.

### 2.1 Data Sources

PubMed (via PubMed.com), MEDLINE (via embase.com) and Embase (via embase.com) were searched systematically using search terminology pertaining to endogenous CS, specifically humanistic (or HRQoL‐related) and economic outcomes. Search algorithms for each electronic database were designed using appropriate search strategies including a combination of medical subject headings (MeSH) or Emtree terms and free texts in titles and abstracts of indexed publications (Tables A1 and A2). This includes terms such as ‘Cushing syndrome’, ‘Cushing disease’, ‘quality of life’ and ‘resource use’ (Tables A1 and A2).

### 2.2 Eligibility criteria

Peer‐reviewed manuscripts published from 2015 to the search date (December 4, 2020) and conference abstracts and posters (subject to data availability and indexed via embase.com) published in the 2 years prior to search date were eligible for inclusion. Only studies published in English were included. Studies focusing on endogenous CS and CD were included and studies focusing on exogenous CS, or any other conditions were excluded from the SLR. Studies eligible for inclusion were primary research studies, economic analyses and models. Previously published SLRs meeting the eligibility criteria were included for reference purposes only. There were no geographical limits on the search. Details of eligibility criteria based on population, intervention, comparator, outcomes and study design criteria (PICOS) are described in Table A3.

### 2.3 Study selection

Upon execution of the systematic searches across the three databases, duplicates were removed using Endnote™, version X9, Philadelphia, PA: Clarivate™ (2013). The screening phase was conducted in 2 steps: title and abstract screening followed by full text screening. The screening of titles and abstracts was conducted by a single reviewer (BO), and a random selection of 15% of excluded citations were validated by a second reviewer (AS). Full text screening was conducted by two independent reviewers (AS and BO), thus ensuring cross‐validation and consensus. Both screening steps were conducted on a web‐based screening software, Covidence (Veritas Health Innovation; Melbourne, Australia). A complete list of included and excluded citations (including the reasons for exclusion) were generated post the full‐text screening phase.

### 2.4 Data extraction

Data from the included studies were extracted in a Microsoft Excel™ (Microsoft Corporation, United States) based evidence grid by one researcher (BO) and validation of the included data were conducted by a second researcher (AS). Detailed data were extracted for included studies such as publication details, study characteristics, patient characteristics and specific QOL and economic burden of illness outcomes, where available.

### 2.5 Outcomes measured

Studies measuring outcomes that impact the HRQoL of patients with CS and the economic impact of CS on patients or the healthcare system were included.

## 3 RESULTS

### 3.1 Study selection

**FIGURE 1:**
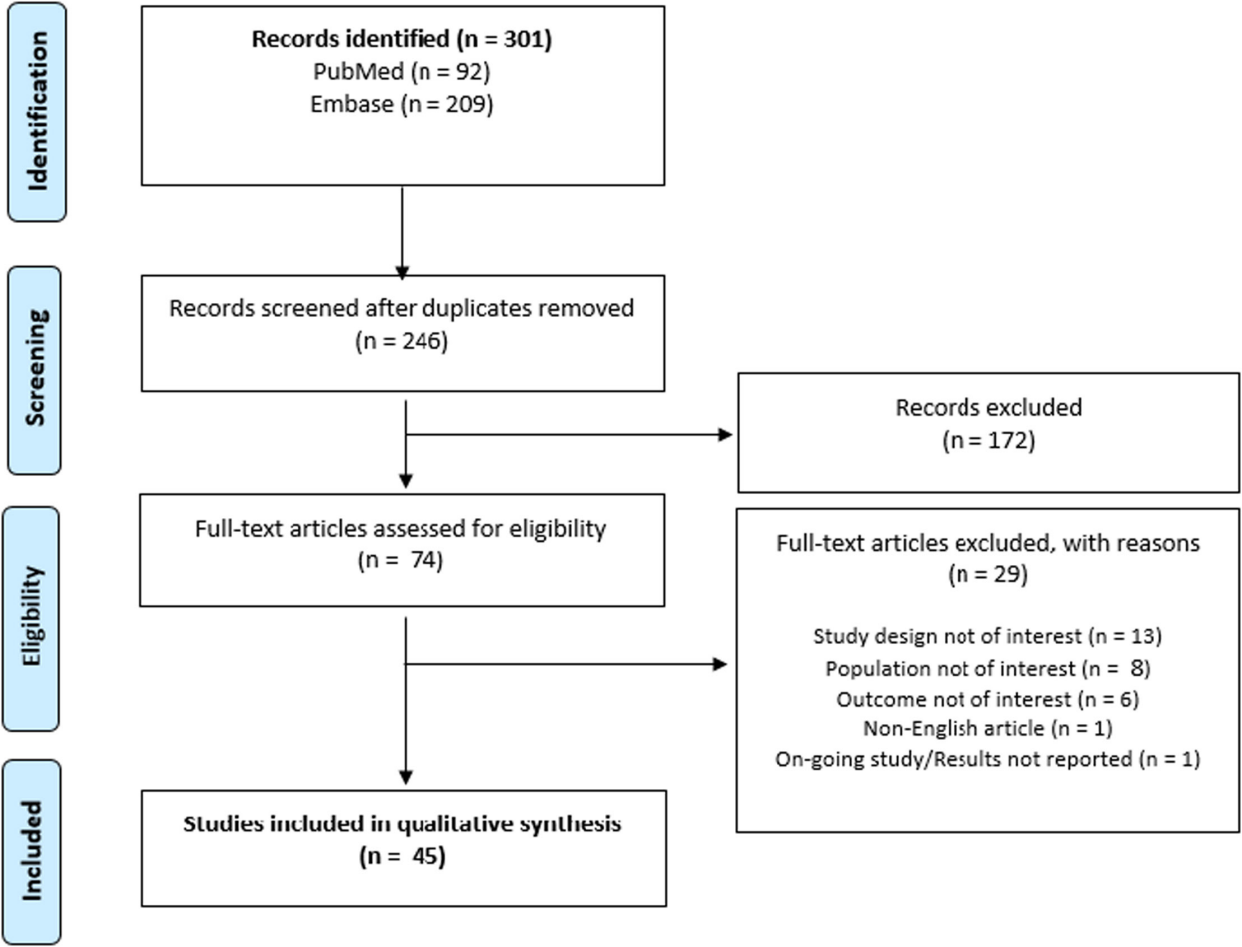
PRISMA diagram. PRISMA: Preferred reporting items for systematic reviews and meta‐analyses.

The searches identified 301 records across the 2 electronic databases, of which 246 (82%) were eligible for screening after deduplication. Following a comprehensive review of the 246 titles and abstracts, 74 (30%) studies were selected for further full‐text review, of which 45 (18% of 246) studies met criteria for qualitative synthesis of the literature (Figure 1).^7^, ^8^, ^12^, ^13^, ^14^, ^15^, ^16^, ^17^, ^18^, ^19^, ^20^, ^21^, ^22^, ^23^, ^24^, ^25^, ^26^, ^27^, ^28^, ^29^, ^30^, ^31^, ^32^, ^33^, ^34^, ^35^, ^36^, ^37^, ^38^, ^39^, ^40^, ^41^, ^42^, ^43^, ^44^, ^45^, ^46^, ^47^, ^48^, ^49^, ^50^, ^51^, ^52^, ^53^, ^54^ Studies were excluded for a variety of reasons throughout the screening process, including but not limited to, the study population, study design or outcomes being out of scope and thus not of interest to the authors (Figure 1).

### 3.2 Study and patient characteristics

Table 1 summarizes the key study and patient characteristics for all included primary studies. Of the 45 studies that met eligibility criteria, 37 (82%) were primary studies and 8 (18%) were previously published SLRs. Data were extracted from the 37 (82%) primary studies and the 8 (18%) SLRs were used for reference purposes only.^7^, ^8^, ^49^, ^50^, ^51^, ^52^, ^53^, ^54^ Of the included studies, 21 (57%) were conducted in Europe, 14 (38%) in North America, 1 in South America (3%) and 1 (3%) in Asia. Among the 37 primary research studies, there were no notable qualitative differences between results in studies conducted in different geographies. Sample sizes of patients with CS ranged from 6 to 1852 patients across the 37 studies. Most studies (30; 81%) reported on prior interventions, including transsphenoidal surgery, adrenalectomy, radiotherapy and pharmacotherapy. Thirty‐two (86%) studies reported HRQoL burden,^12^, ^13^, ^14^, ^18^, ^19^, ^20^, ^21^, ^22^, ^23^, ^24^, ^25^, ^26^, ^27^, ^28^, ^29^, ^30^, ^33^, ^34^, ^35^, ^36^, ^37^, ^38^, ^39^, ^40^, ^41^, ^42^, ^43^, ^44^, ^45^, ^46^, ^47^, ^48^ 4 (11%) studies reported economic burden^15^, ^16^, ^17^, ^27^ and one (3%) study reported both.^25^ Eight (22%) studies reported pre−/post‐surgery outcomes.^18^, ^19^, ^37^, ^38^, ^42^, ^43^, ^47^, ^48^ Thirty‐one (84%) studies reported HRQoL using validated patient reported outcome (PRO) measures.^15^, ^16^, ^19^, ^21^, ^23^, ^24^, ^25^, ^26^, ^27^, ^28^, ^29^, ^30^, ^31^, ^32^, ^33^, ^34^, ^35^, ^36^, ^37^, ^38^, ^39^, ^40^, ^41^, ^42^, ^43^, ^44^, ^45^, ^55^, ^56^, ^57^ A comprehensive list of the PRO measures used to measure the outcomes was recorded (Table A4). Of the 31 (84%) studies, 10 (32%) used 1 PRO measure, 7 (23%) used 2 different PRO measures, 6 (19%) used 3 different PRO measures and 6 (19%) used 5 or more different PRO measures. CushingQoL was the most commonly used PRO measure across 18 (58%) studies followed by short form (SF) questionnaires (SF‐36, SF‐12 and SF‐HRQoL).

**TABLE 1:** Key study and patient characteristics.

Study characteristics			Patient characteristics			
Author, year	Study design	Sample size	Treatment cohort	Age (years) Mean ± [SD]	Female %	Prior treatments
Andela, 2015^12^	Prospective cohort study	6	Cushing's Disease	NR	67%	1. Radiotherapy ±17%, Transsphenoidal surgery ±83% 2. Current suppressant medication ±67%
Broder, 2015^16^	Retrospective cohort study	1852	Cushing's Disease	42.9 ± 12.3	78%	NR
Broder, 2015^15^	Retrospective cohort study	685	Cushing's disease	41.7 ± 13.4	81%	1. Pituitary surgery ±24.4%, adrenalectomy ±1.9%, dopamine agonists ±7%, ketoconazole±7%, mitotane ±0.1%, radiotherapy ±3.5%
Milian, 2015^30^	Retrospective cohort study	176	Cushing's DISEASE	46.1 ± 13.7	18%	1. Surgery 2. Hydrocortisone replacement therapy ±52.3%, growth hormone ±8.0%, thyroid hormones ±39.2%, sex hormones ±14.2%
Papakokkinou, 2015^36^	Cross‐sectional study	51	Patients in remission	NR	NR	Surgery
			Control	NR	NR	N/A
Andela, 2016^13^	Prospective cohort study	72	Cushing's disease	54.5 ± 12.6	78%	1. Pituitary surgery ±74% 2. Radiotherapy ±31% 3. 21 patients diagnosed with adrenal CD 57% treated with bilateral adrenalectomy, 47.6% treated with unilateral adrenalectomy and 68% treated with hormonal replacement therapy and/or suppressant medication
Burton, 2016^17^	Retrospective cohort study	877	Cushing's disease	42 ± 14	75%	1. Hypophysectomy, radiation, bilateral inferior petrosal sinus sampling
Martinez‐Momblan, 2016^29^	Prospective cohort study	30	Control Group‐Cushing's disease	48.3 ± 13.2	41%	1. Radiotherapy 2. medical therapy with ketoconazole and metyrapone
		31	Intervention‐Cushing's disease	46.1 ± 12.2	43%	1. Radiotherapy 2. medical therapy with ketoconazole and metyrapone
Nader, 2016^33^	Retrospective cohort study	54	In remission	48.0 ± 15.5	NR	1. Neurosurgery
			Active Cushing's disease	48.0 ± 15.5	NR	1. Neurosurgery
Papoian, 2016^37^	Cross‐sectional study	267	Cushing's disease	48 ± 12	90%	1. Pituitary surgery ±68%, adrenalectomy ±32.9%, radiation ±18%
Siegel, 2016^39^	Cross‐sectional study	176	Cushing's disease	46.1 ± 13.69	82%	1. 1–2 Pituitary surgeries ±69.2% 3 Pituitary surgeries ±19.9%, 4 Pituitary surgeries ±9.6%, radiotherapy ±13.6%
Tiemensma 2016^40^	Retrospective cohort study	332	All patients	48.41 ± 13.1	89%	1. 68% Transsphenoidal surgery, 7% received postoperative radiotherapy,19% underwent unilateral adrenalectomy and 17% underwent bilateral adrenalectomy
Ye 2017^47^	Retrospective cohort study	51	All patients	42.4	NR	NR
DeBucy, 2017^20^	Prospective cohort study	206	Pituitary Cushing's syndrome	48.2 ± 14.5	NR	NR
Valassi, 2017^41^	Cross‐sectional study	25	Cushing's syndrome in remission	48.8 ± 11.8	89%	1. Transsphenoidal surgery ±69% 2. Radiotherapy ±24%, previous medical treatment ±56%
Ehrlich, 2018^21^	Prospective cohort study	17	Endogenous Cushing's syndrome	NR	76%	1. Surgical intervention
Keil, 2018^24^	Prospective cohort study	64	Cushing's Disease	12.6 ± 3	53%	NR
Kritschmann‐Andermahr, 2018^25^	Cross‐sectional study	148	US patients	50.1 ± 13.73	92%	NR
			German patients	48.8 ± 13.64	83%	NR
Lee, 2018^27^	Retrospective cohort study	89	Endogenous Cushing's syndrome	NR	NR	NR
Lobatto, 2018^28^	Cross‐sectional study	32	Cushing's disease	NR	NR	NR
Mueller, 2018^32^	Prospective cohort study	107	Endogenous Cushing's syndrome	NR	74%	1. Underwent conventional/strain imaging echocardiography
Osswald, 2018^35^	Cross‐sectional study	119	Ectopic Cushing's syndrome	NR	59%	NR
			Cushing's disease	NR		NR
Valassi 2018^42^	Retrospective cohort study	410	All patients baseline	NR	83%	NR
			Pituitary Cushing's syndrome baseline	NR	83%	NR
			Adrenal Cushing's syndrome baseline	NR	83%	NR
			All Patients first preoperative	NR	83%	NR
			Pituitary Cushing's syndrome first pre‐operative	NR	NR	NR
			Adrenal Cushing's syndrome first pre‐operative	NR	NR	NR
			Pituitary Cushing's syndrome last follow‐up	NR	NR	NR
			Adrenal Cushing's syndrome last follow‐up	NR	NR	NR
			Pituitary Cushing's syndrome remission	NR	NR	NR
			Adrenal Cushing's syndrome remission	NR	NR	NR
Valassi, 2018^43^	Prospective cohort study	413	Pituitary Cushing's syndrome	NR	NR	NR
			Adrenal Cushing's syndrome	NR	NR	1. Transsphenoidal surgery ±67%, bilateral adrenalectomy ±10%, unilateral adrenalectomy ±21%
Vega‐Beyhart 2018^44^	Cross‐sectional study	30	Cushing's disease	NR	100%	NR
Vega‐Beyhart, 2018^45^	Cross‐sectional study	30	Cushing's disease	NR	NR	NR
Vermalle 2018^46^	Retrospective cohort study	63	Cushing's syndrome	NR	87%	1. Transsphenoidal surgery ±67%, bilateral adrenalectomy ±10%, unilateral adrenalectomy ±21%
Chen, 2019^18^	Prospective cohort study	49	Post‐traumatic stress syndrome	34.7 ± 10.4	68%	NR
Fleseriu, 2019^22^	RCT	104	All patients	42.5 ± 13.1	81%	1. Surgery ±80.8% 2. Pituitary irradiation ±26%
			600 μg pasireotide	45.5 ± 13.1	75%	1. Surgery ±77.6% 2. Pituitary irradiation ±24.5%
			900 μg Pasireotide	39.9 ± 12.6	85%	1. Surgery ±83.6% 2. Pituitary irradiation ±27.3%
Nankova, 2019^34^	Cross sectional study followed by prospective cohort study	160	Active disease	47.4 ± 13	91%	1. Adrenalectomy
			Active disease‐pituitary	47.4 ± 13	91%	1. Adrenalectomy
			Active disease‐adrenal	47.4 ± 13	91%	1. Adrenalectomy
			Remission	47.3 ± 11.29	85%	1. Adrenalectomy
Sarkis, 2019^38^	Retrospective cohort study	34	BADX	50 ± 14.8	82%	NR
			Non‐BADX	48.5 ± 16	71%	NR
Zarino, 2019^48^	Prospective cohort study	10	Cushing's disease	47.9 ± 12.5	60%	1. Transsphenoidal surgery ±100%
Bauduin, 2020^14^	Prospective cohort study	25	Cushing's disease	45 ± 8	84%	1. Transsphenoidal surgery
Cusimano, 2020^19^	Retrospective cohort study	96	Treated validation group 1‐post transsphenoidal surgery	46.44	90%	1. Radiation GKRS ±5% 2. Ketoconazole ±5% 3. Bromocriptine, cabergoline, quinagolide ±2.5% 4. Hormone therapy ±25%, other ±5%
			Treated validation group 2 post transsphenoidal surgery	43.44	20%	1. Ketoconazole ±12% 2. Bromocriptine, cabergoline, quinagolide ±4%, hormone therapy ±12%
Geer, 2020^23^	RCT	77	Endogenous Cushing's syndrome	44.1 ± 12.9	84%	1. Medical therapy
Lacroix, 2020^26^	RCT	150	Cushing's disease	38.5	79%	NR
Moyers, 2020^31^	Retrospective cohort study	147	Cushing's syndrome	51.71 ± 13.08	95%	1. Transsphenoidal surgery ±74.1%, adrenal surgery on one side ±2.4%, bilateral adrenal surgery ±10%, post‐operative radiotherapy ±7.5%, other ±6.1%
			Cushing's disease	51.71 ± 13.08	95%	1. Transsphenoidal surgery ±74.1%, adrenal surgery on one side ±2.4%, bilateral adrenal surgery ±10%, post‐operative radiotherapy ±7.5%, other ±6.1%

Abbreviations: BADX, bilateral adrenalectomy; N/A, not applicable; NR, not reported; RCT, randomized controlled trial; SD, standard deviation.

In the 29 (78%) studies reporting patients' sex, female patients comprised 18%–100% of the study cohorts. In the 26 (70%) studies reporting patient age, the mean age ranged from 13 to 55 years. Seven studies (19%) reported on comorbidities associated with CS, including diabetes, hypertension, depression, osteoporosis and obesity.^15^, ^17^, ^26^, ^35^, ^38^, ^46^, ^48^

### 3.3 Cushing's syndrome specific HRQoL

CS specific HRQoL was evaluated in 18 (49%) studies using three different PRO measures—CushingQoL questionnaire, Tuebingen Cushing's Disease Quality of Life Inventory (Tuebingen CD‐25) and QoL‐Cushing's disease (QoL‐CD).^19^, ^22^, ^23^, ^24^, ^26^, ^29^, ^30^, ^31^, ^33^, ^34^, ^35^, ^36^, ^37^, ^38^, ^39^, ^40^, ^42^, ^43^, ^46^ CushingQoL is a 12‐item, CS‐specific HRQoL PRO that examines patients' HRQoL over the past 4 weeks.^8^ The Tuebingen CD‐25 is a multidimensional HRQoL PRO that examines six domains (mood, sexual activity, eating behaviour, social environment, physical and cognitive functions).^30^ QoL‐CD assesses disease progression and evaluates medical treatment alongside other HRQoL attributes such as general, emotional and mental health.^19^ These PRO measures incorporated disease specific questions focused on psychological, social and physical issues that can impact a patient's day to day life. CushingQoL was the most commonly utilized PRO measure (Table 2). Using this measure, for which higher scores are associated with improved overall QoL, mean total scores ranged from 27 to 71 (out of 100).^22^, ^23^, ^26^, ^29^, ^30^, ^37^, ^39^, ^42^, ^43^ CushingQoL scores improved in patients with remission, post‐surgery or post‐pharmacotherapy compared to those with active disease, pre‐surgery and pre‐pharmacotherapy, respectively. There was an overall improvement in total CushingQoL scores that cannot be attributed to a singular domain. However, this improvement was not always consistent.^23^, ^37^, ^42^

**TABLE 2:** Studies reporting CS specific HRQoL on CushingQoL.

Author, year	Patient cohort	Sample size	Interventions	Scores	Mean ± SD
Longitudinal time point analysis					
Valassi, 2018^42^	Active CS, baseline	261	Pituitary surgery	Total score	41 ± 18
	Active CS, last follow‐up visit	144		Total score	56 ± 20
	Active CS, first post‐operative visit	136		Total score	54 ± 20
Papoian, 2016^37^	Endogenous CS, < 3‐month follow‐up	133	Pituitary surgery/adrenalectomy/radiation	Total score	38 ± 22
	Endogenous CS, < 6‐month follow‐up	59		Total score	45 ± 23
	Endogenous CS, <1 year follow‐up	24		Total score	47 ± 19
	Endogenous CS, < 2‐year follow‐up	4		Total score	34 ± 25
	Endogenous CS, 2–5 year follow‐up	3		Total score	49 ± 16
	Endogenous CS, >5‐year follow‐up	1		Total score	71
Milian, 2015^30^	CD	176	1. Surgery 2. Hydrocortisone replacement therapy, growth hormone, thyroid hormone, sex hormone	Total score	58.1 ± 17.9
B Remission status					
Papoian, 2016^37^	Endogenous CS, achieved remission^a^	195	Pituitary surgery/adrenalectomy/radiation	Total score	48 ± 22
	Endogenous CS, currently in remission^a^	193		Total score	48 ± 22
	Endogenous CS, 0–2 Years in remission^a^	45		Total score	51 ± 20
	Endogenous CS, 2–5 Years in remission^a^	42		Total score	53 ± 22
	Endogenous CS, 5–10 years in remission^a^	24		Total score	50 ± 24
	Endogenous CS, 10+ years in remission^a^	19		Total score	55 ± 23
	Endogenous CS, has not Achieved remission^a^	69		Total score	27 ± 16
	Endogenous CS, not currently in remission^a^	67		Total score	27 ± 17
C Pharmacotherapy					
Lacroix, 2020^26^	CD	150	Pasireotide	Total score	40.3 ± 17.5
Fleseriu, 2019^22^	Active CD, Week 12	104	Pasireotide	Total score	67.1 ± 104.3^b^
	Active CD, Week 24	104		Total score	82.3 ± 139.4^b^
	Active CD, Week 48	104		Total score	34.4 ± 49.9^b^
Geer, 2020^23^	Endogenous CS, baseline	74	Levoketoconazole	Total score	44.3 ± 21.3
	Endogenous CS, month 3	61	Levoketoconazole	Total score	50.9^c^
	Endogenous CS, month 6	54	Levoketoconazole	Total score	53.9^c^

Abbreviations: CD, Cushing's disease, CS, Cushing's syndrome, NR, not reported; SD, standard deviation.

^a^ After diagnosis of CS.

^b^ Mean % change from baseline.

^c^ Mean change from baseline.

*Note*: Table includes data for only selected studies that used CushingQoL.

There was an increase in mean CushingQoL scores from baseline to follow‐up after treatment with levoketoconazole.^23^ Of the 60 patients evaluated in this study, 40% met or exceeded the minimally important difference (MID) of 10.1 points for CushingQoL at Month 3, and 47% of the 51 patients met the MID at Month 6.^23^ Additionally, one study (3%) reported that mean CushingQoL scores increased from baseline by 67% at Week 12, to 82.3% at Week 24, and 34.4% at Week 48, exceeding the minimal important difference (MID) threshold after treatment with pasireotide.^22^

### 3.4 Psychological and emotional health

#### 3.4.1 Depression and anxiety

Results of 8 (26%) studies reporting depression or anxiety using PRO measures are summarized in Table 3.^14^, ^23^, ^33^, ^38^, ^39^, ^41^, ^46^, ^48^ One study (9%) reported mean depression scores (± standard deviation (SD)) using the Minnesota Multiphasic Personality Inventory (MMPI‐II), a self‐reported psychometric questionnaire, for patients with CD and found that both preoperative (59.7 ± 5.0) and 12 months postoperative (63.9 ± 3.6) scores were below the threshold (≥65) indicating a clinical diagnosis of depression.^48^ Using the Hospital Anxiety and Depression Scale (HAD/HADS‐D), a 21‐point scale in which scores of 11 or higher indicate depression and anxiety, 1 (9%) study reported mean depression and anxiety scores (5.5 and 7) among patients with CD who underwent pituitary surgery.^46^ Similarly, using the Freiburg questionnaire on the use of coping strategies (FKV‐LIS), a scale with scores ranging from 1 to 5 where higher scores indicate more effective use of coping strategies, another study (9%) reported a mean score of 2.3 among patients with CD post pituitary surgery.^39^, ^58^ Of the 10 (27%) studies that evaluated depression or anxiety, 5 (50%) studies reported depression using Beck Depression Inventory (BDI I/II), a depression‐specific PRO measure that evaluates the severity of depressive symptoms on a scale ranging from 0 to 63, where higher scores indicate more severe depression and a mean change of ≥3 BDI‐II points indicates a clinically meaningful change in depression due to the treatment.^23^, ^33^, ^38^, ^41^, ^48^, ^59^, ^60^ In a single study (10%) reporting scores for patients with active CS at baseline and 6 months post‐maintenance phase of levoketoconazole treatment, the mean scores were 17.1 and 12.5, respectively, indicating the presence of minimal depression but a meaningful improvement in depression scores post‐treatment given that they exceeded the MID threshold.^23^ In another study (10%), the mean total BDI‐II score reported in patients with CS in remission (11.3 ± 10.2) was higher than healthy controls (3.4 ± 2.9) indicating greater depressive symptoms in patients in remission compared to healthy controls.^41^ Finally, 1 (10%) study comparing patients in remission (*n* = 44) to those with active disease (*n* = 8) using the BDI, reported that a lower proportion of patients in remission had depressive symptoms relative to those with active disease across depression severity levels: mild to moderate (20% vs. 50%) and severe depression (22% vs. 25%), respectively.^33^

**TABLE 3:** Studies measuring depression or anxiety using PROs.

Author year	Sample size	PRO	Patient cohort	PRO score reported as	Scores
Depression					
Zarino, 2019^48^	10	BDI‐II	Pre‐transsphenoidal surgery	Mean ± SD	15.4 ± 8.57
			Post‐transsphenoidal surgery	Mean ± SD	9.7 ± 3.06
		MMPI‐II	Pre‐transsphenoidal surgery	Mean ± SD	59.66 ± 5
			Post‐transsphenoidal surgery	Mean ± SD	63.9 ± 3.57
Vermalle 2018^46^	63	HAD	Post‐surgery/radiation/pharmacotherapy^c^	Median (IQR)	5.5 (4.0–9.0)
Valassi, 2017^41^	36	BDI‐II	Total score^c^	Mean ± SD	11.3 ± 10.2
			Affective dimension^c^	Mean ± SD	6.7 ± 1.3
			Somatic dimension^c^	Mean ± SD	4.6 ± 0.5
Siegel, 2016^39^	176	FKV‐LIS	Patient with CD post‐pituitary surgery	Mean ± SD	2.3 ± 0.85
		HADS‐D	Patient with CD post‐pituitary surgery	Mean ± SD	5.5 ± 4.45
Sarkis, 2019^38^	17	BDI	BADX	Median (IQR)	16 (9.5–24)
			Non‐BADX	Median (IQR)	9 (4.0–14.0)
Nader, 2016^33^	46	BDI	No depression^c^ ^,^ ^b^	Proportion	55%
			Mild to moderate depression^c^ ^,^ ^b^	Proportion	20%
			Severe depression^c^ ^,^ ^b^	Proportion	22%
	8	BDI	No depression^c^ ^,^ ^b^	Proportion	25%
			Mild to moderate depression^c^ ^,^ ^b^	Proportion	50%
			Severe Depression^c^ ^,^ ^b^	Proportion	25%
	59	BDI‐II	Total score‐baseline	Mean ± SD	17.1 ± 12.9
Geer, 2020^23^	52	BDI‐II	Total score‐baseline to month 3^a^	Mean ± SD	14.4
	42	BDI‐II	Total score‐baseline to month 6^a^	Mean ± SD	12.5
Hysteria					
Zarino, 2019^48^	10	MMPI‐II	Pre‐transsphenoidal surgery	Mean ± SD	49.11 ± 7.54
		MMPI‐II	Post‐transsphenoidal surgery	Mean ± SD	52.7 ± 6.09
Anxiety					
Vermalle 2018^46^	63	HAD	Post‐surgery/radiation/pharmacotherapy^c^	Median (IQR)	8 (5.0–11.0)
Siegel, 2016^39^	176	HADS‐D	Patients with CD post‐pituitary surgery	Mean ± SD	7 ± 4.27
Bauduin, 2020^14^	25	BAI	Total score^d^	Mean ± SD	28.4 ± 17.4

Abbreviations: BAI, beck anxiety inventory; BDI, beck depression inventory; BDI‐II, beck depression inventory‐II; FKV‐LIS, freiburg questionnaire of the use of coping strategies; HAD, hospital anxiety and depression; MMPI‐II, minnesota multiphasic personality inventory 2; PROs, patient‐reported outcome measures.

^a^ Post pharmacotherapy.

^b^ Post neurosurgical treatment.

^c^ Patients in remission.

^d^ Long‐term remitted Cushing's disease patients scoring ranges for BDI‐I/II and BAI (0 = no depression/anxiety to 63 = severe depression/anxiety), MMPI‐II (clinical cut off ≥65 indicated the presence of depression) and HAD (0–7 = no depression, 8–10 = likely depression, >11 = depression).

*Note*: Table includes data for only selected studies measuring depression or anxiety.

#### 3.4.2 Non‐specific PRO measures

General HRQoL assessed the patients' perceived physical and mental health over time using validated generic (not specific to endogenous CS) PRO measures. Of the 31 (84%) studies that reported HRQoL, 21 (68%) reported general HRQoL using SF questionnaires (SF‐36, SF‐HLQ, SF12v2Score) (*n* = 15; 71%), EuroQoL version 5D (EQ‐5D) (*n* = 4;19%), Work Role Functioning Questionnaire (WRFQ) (*n* = 1; 5%) and positive and negative affect schedule (PANAS) (*n* = 1; 5%).^12^, ^18^, ^19^, ^20^, ^21^, ^25^, ^26^, ^29^, ^30^, ^36^, ^37^, ^38^, ^39^, ^41^, ^42^, ^43^, ^44^, ^45^, ^47^, ^48^ The SF‐36 questionnaire (total score ranging from 0 = maximum disability to 100 = no disability) was the PRO measure utilized most frequently to report general HRQoL (*n* = 14; 67%). Of the 14 (67%) studies, 8% or 57% (7 studies conducted in Europe and 1 in the US; 88% and 13%, respectively) reported mean, post‐treatment physical functioning scores ranging from 48 to 80.^28^, ^29^, ^30^, ^33^, ^38^, ^39^, ^47^, ^48^ Using SF‐36, another study (7%) reported significantly worse mean QoL scores in females compared to male patients with active disease in the following domains: physical role functioning (51.8 ± 43.1 vs. 73.4 ± 36.4), emotional role functioning (61.2 ± 43.9 vs. 79.6 ± 37.2), vitality (44.5 ± 19.7 vs. 51.5 ± 19.8) and mental health (61.2 ± 21.3 vs. 68.1 ± 19.9).^39^

Three (10%) studies reported cognitive, psychological or emotional HRQoL using the Coping Orientation to Problems Experienced Inventory (Brief‐COPE), bern embitterment inventory (BEI) and FKV‐LIS and MMPI‐II PRO measures, among others.^13^, ^36^, ^39^ Three (10%) studies reported fear or panic using the Fear Questionnaire (FQ) or MMPI‐II.^12^, ^14^, ^48^ In one study (3%) reporting total fear scores for patients with CD in long‐term remission (24.5 out of 120) and matched controls (14.2 out of 120), the difference between the two groups was more than 10 points, indicating a clinically relevant difference on the fear psychopathology subscales.^14^ A qualitative study (3%) reported that both active and remitted patients with CD have persistent thoughts, fear of collapsing and fear of recurrence.^12^ Lastly, one study discussing qualitative findings reported that CD patients reported paranoia and hypochondriasis that persisted post operatively.^48^

### 3.5 HRQoL and mood: Pre‐ and post‐treatment

Eight studies (22%) evaluated pre‐and post‐surgery HRQoL outcomes.^18^, ^19^, ^36^, ^38^, ^42^, ^43^, ^47^, ^48^ Among these 8 (22%) studies, 4 (50%) had longitudinal follow‐up of greater than or equal to 1‐year post‐surgical procedure.^18^, ^37^, ^42^, ^48^ Four studies (50%) reported HRQoL outcomes using CS specific PRO measures, 2 (25%) reported HRQoL using SF‐36 and, 2 (25%) used BDI‐II and MMPI. Of the 8 (22%) studies, 6 (75%) reported statistically significant improvement in HRQoL following surgical intervention.^18^, ^19^, ^37^, ^38^, ^43^, ^48^

Two (25%) studies reported SF‐36 scores from 1 to 6.1 years post‐surgery with statistically significant improvements in bodily pain (change from baseline ranged from 3% to 40%), general health (change from baseline ranged from 17% to 37%), and social functioning (change from baseline ranged from −4% to 50%).^38^, ^48^ One study (13%) demonstrated directionally improved CushingQoL total scores at the first post‐operative visit compared to baseline (56 at first postoperative visit vs. 41 at baseline, with higher scores associated with improved CushingQoL); statistical significance was not analysed.^42^ Using a 3‐point scale in which 1 is the best possible and 3 is the worst possible HRQoL, a second study reported significantly improved QoL‐CD mean scores in emotional health (1.93 vs. 2.25) and physical health (1.74 vs. 2.17) post‐ vs pre‐transsphenoidal surgery, respectively.^19^

One study (13%) reported mean CushingQoL scores of patients who received 1, 2, 3 or 4+ pituitary surgeries (41 ± 22, 45 ± 23, 35 ± 21 and 52 ± 20). No statistically significant differences were found in CushingQoL scores between these cohorts.^37^

Two studies (25%) using MMPI‐II and BDI reported post‐surgery depression scores.^38^, ^48^ MMPI‐II scores increased 12 months after surgery from 59.66 to 63.90; these scores did not meet the threshold for a clinical diagnosis of depression (≥65).^38^, ^48^ BDI scores were significantly higher in the pre‐surgery period than the post‐surgery period (15.40 compared to 9.70) (*p* < .05), indicating significantly worse depression pre‐surgery.^46^ In another study (13%), BDI scores were also significantly higher when comparing patients who received bilateral adrenalectomy with patients who received non‐surgical treatments (16 compared to 9) (*p* < .05), signifying heightened depression in the post‐surgery patients.^36^ The remission status was only confirmed in one of the two studies.^38^ Another study (13%) reported HRQoL outcomes pre‐ and post‐pharmacotherapy (pasireotide) using CushingQoL. Mean HRQoL scores improved from baseline through Week 12 and were sustained until Week 24 but declined at Week 48 compared to baseline. These changes in the CushingQoL scores exceeded the MID threshold.^22^

### 3.6 Other types of HRQoL burden

Fatigue was reported in 1 (3%) study using the Mental Fatigue Scale (MFS‐Score) PRO measure and in another study using qualitative reporting by patients with CS.^36^ The MFS questionnaire response set consists of 4 alternatives where a rating of 0 reflects normal function, 1 indicates a problem, 2 indicates pronounced symptom and 3 indicates a maximal symptom.^36^ One study (3%) using MFS‐Score reported statistically significant differences between patients with CD in remission and healthy controls for fatigue (1.25 vs. 0.5), mental fatigue (1.25 vs. 0.5), mental recovery (1 vs. 0.5), irritability (0.8 vs. 0.5), light sensitivity (0.5 vs. 0.25) and decreased sleep at night (1 vs. 0.5). A second study reported that patients with diagnosed CD, either active or in remission, experienced both physical and mental fatigue. This corroborates the findings of the first study suggesting patients with CD experience more fatigue than healthy controls and highlights the unmet clinical need in these patients.^12^

### 3.7 Economic burden

Five studies (14%) reported on economic burden for patients with CS, including 3 (60%) studies reporting direct economic burden (including costs directly related to healthcare expenditure, e.g. medical costs, pharmacy costs and inpatient hospital costs) and 2 (40%) studies reporting both direct and indirect burden (including direct costs and costs that are not directly related to healthcare, e.g. cost to the patient to travel to receive care).^15^, ^16^, ^17^, ^25^, ^27^ One study (20%) reported that the all‐cause, total per patient per month (PPPM) cost for a CD patient was $3232 as measured by administrative claims from a large health insurance database. The majority of PPPM costs ($2800) were direct medical costs including inpatient hospital stays, outpatient and emergency room visits, and ambulatory services use.^17^ Two studies (40%) reported that the mean total annual healthcare system costs associated with CD were $26,269 and $34,992, respectively (SD not reported), with non‐pharmacy costs making up most of these costs ($21,704 and $31,395).^15^, ^16^

## 4 DISCUSSION

To the authors' knowledge, this systematic review is the most up‐to‐date and comprehensive qualitative synthesis of scientific evidence published on both the HRQoL and economic burden of patients with CS. It is possible that other studies of interest have been published as the search date for this SLR. In accordance with the published literature, our findings demonstrate that patients with endogenous CS experience a substantial, complex and multi‐symptomatic HRQoL burden. Even though the HRQoL of patients with CS improves post‐treatment, it does not normalize and may require further intervention. Additionally, when comparing patients with CS to healthy populations across specific domains, such as depression symptoms, patients with CS have worse outcomes indicating that these symptoms are not fully addressed by the current care paradigm.

Patient wellbeing is impacted by a high HRQoL burden, which persists in key domains despite current multi‐modal treatment interventions. The burden of illness described in this manuscript relies heavily on the use of PRO measures. By providing previously undocumented CushingQoL reference values for a variety of endogenous CS disease and treatment scenarios, this review may serve as a potential resource to guide clinicians interpreting the CushingQoL in research studies and clinical care settings. Even when disease‐specific PRO measures such as the CushingQoL and Tuebingen‐25 are used in studies examining PROs, there is often still a gap in measuring depressive and cognitive symptoms.^53^

Of the 31 studies examined in this SLR, 61% utilized 1 or more PRO measures. Combining a CS specific PRO measure with a generic PRO measure (e.g. the SF questionnaires) allows a patient to not only be compared to other patients with CS, but also to patients with other diseases or healthy population norms. While this provides a considerably broader view of overall HRQoL, currently available validated PRO measures do not thoroughly assess the symptom‐specific burdens related to muscle atrophy and weakness, fatigue and pain among patients with CS. Even though some of the PRO measures evaluate physical health as a domain, these studies primarily reported an overall score and did not report physical functioning domain scores. This denotes an unmet need for the assessment and reporting of the physical function burden associated with CS via validated PROs.

In line with a previously published SLR, this review found substantial differences between the overall HRQoL of healthy individuals and patients with CS in remission.^51^ The mean EQ‐5D VAS score (range of 0–100, with higher score correlating to improved HRQoL) for patients with CS was 54 at baseline, 66 at first postoperative visit and 69 in longer‐term remission.^42^ The range of mean normative EQ‐5D VAS scores (75–84) reported across European countries (including Belgium, Denmark, France, Germany, Greece, Hungary, Italy, Netherlands, Slovenia, Spain and Sweden) indicated that endogenous CS patients continue to have worse HRQoL scores compared to healthy individuals, suggestive of long‐term effects of CS.^43^, ^61^ Similarly, CS had a substantial adverse impact on the physical function dimension in patients, relative to UK population norms.^62^

This study reported key findings on HRQoL and mood pre‐ and post‐treatment. One study, focused on CD patients who did not achieve remission, reported a worsened depression score 1‐year post‐transsphenoidal surgery.^46^ A second study examined CD patients in remission, and reported worse depression scores among CD patients an average of 8.4 years following bilateral adrenalectomy, compared to CD patients in remission for an average of 2.7 years who had not undergone bilateral adrenalectomy.^36^ However, of the eight studies that evaluated pre‐ and post‐surgery outcomes, six studies demonstrated an overall HRQoL improvement post‐surgery.^18^, ^19^, ^37^, ^38^, ^43^, ^48^ Although, in other published SLRs, when evaluating specific subdomains of each HRQoL questionnaire including anxiety, depression and fear, patients reported worsened scores potentially due to cortisol withdrawal or other disease factors.^49^, ^53^, ^54^, ^63^, ^64^, ^65^ Additionally, comorbidities impacted overall QoL for CS patients even post‐treatment.^66^ A clinically relevant aspect of the patient's treatment experience is the onset of cortisol withdrawal symptoms following rapid decreases in circulating cortisol levels after prolonged exposure. Cortisol withdrawal is a well‐described constellation of symptoms experienced by patients with CS, particularly following surgical remission, but also in those receiving medical therapy.^49^, ^53^, ^54^, ^63^, ^64^, ^65^ Cortisol withdrawal symptoms after treatment, including fatigue, nausea, body pain and mood changes are well documented clinically; however confirmation of the role of these symptoms, and post‐surgical recuperation, in long‐term HRQoL is needed. These symptoms further complicate the management of CS and highlight the need for longitudinal research using PROs in this population. Additionally, surgical interventions may not necessarily correlate with remission leading to a unidirectional improvement in HRQoL. In accordance with published literature, this SLR shows that despite surgical interventions, patients with CS may not achieve HRQoL similar to population norms, suggesting that these interventions are not truly ‘curative’. Lack of significant improvement post‐operatively suggests additional interventions may be needed to restore normal HRQoL for patients even after successful treatment of endogenous CS.^49^, ^53^, ^54^, ^63^, ^64^, ^65^

While several studies in the past 5 years reported HRQoL pre‐and post‐surgery, no study has reported HRQoL outcomes pre‐ and post‐radiotherapy and few studies report on the progression of HRQoL pre‐ and post‐pharmacological interventions. CushingQoL scores were found to exceed the MID threshold of ≥10.1 from baseline to follow‐up after pharmacotherapy with levoketoconazole and pasireotide, suggesting improvements in QoL with treatment.^22^, ^23^ Notably, compared to patients with active disease, QoL was found to be better in patients in remission across all interventions.^37^ However, both groups of patients with CS still had worse HRQoL than their counterparts without CS.^37^ This confirms previous key findings that patients with active CS have substantially lower QoL scores than those in remission, regardless of sex, age or time in remission, and emphasizes the importance of attaining biochemical control of cortisol production. Further research is needed to understand the benefits and limitations of using pharmacotherapy for cortisol lowering, and to identify those who are most likely to benefit from it.

This study also aimed to describe sex differences in the presentation of disease burden. The prevalence of CS is higher in female compared to male patients. The studies reporting HRQoL and sex association data demonstrate worse HRQoL in females compared to males within subdomains of SF‐36 scores, specifically physical role functioning, emotional role functioning, vitality and mental health.^35^, ^39^ However, conclusions about sex differences in HRQoL are limited due to small study sample sizes and thus additional research is needed to improve our understanding of sex specific differences in HRQoL in CS, and to better describe the burden in male patients.^35^, ^39^

Patients with CS not only experience a significant HRQoL burden throughout their patient journey, but they also experience economic burden at individual and systemic levels. Although previous SLRs have examined various aspects of the burden of CS, few have described the economic burden of the disease and none have highlighted societal costs related to lost work or decreased productivity due to impairment.^8^, ^15^, ^16^, ^17^, ^25^, ^27^, ^50^, ^51^, ^53^, ^54^ Among the two studies reporting total annual healthcare expenditures, costs ranged from $26,269 to $34,992; however, costs associated with prior treatments and office visits incurred while seeking a definitive diagnosis were not reported. Therefore, it is likely that the overall economic burden on patients with CS is much greater than what is reported in the current literature. By comparison, in 2020, the average per person US healthcare expenditure was found to be $12,530, underscoring the high cost burden of disease in patients with CS.^67^ The limited availability of economic data coupled with limited information on how the existing economic evidence was compiled, highlights the lack of a holistic economic burden analysis. Future economic burden studies should not only evaluate the annual long‐term economic burden incurred by patients, but also how that economic burden is impacted by delays in diagnosis and impairments in long‐term HRQoL, despite successful treatment.

### 4.1 Limitations of this SLR

This SLR has several limitations to note. In most of the included studies, the analysis was conducted only in patients with CS and the studies did not include a normative population comparator. Published manuscripts and conference abstracts included in this review were given equal weighting in the qualitative synthesis.

Furthermore, studies published on the drug osilodrostat are not captured in this study. Though the drug was approved by the FDA in March 2020, studies reporting on the impact of osilodrostat on patients' disease burden were not published by the search date. It will be important for future SLRs capturing the disease burden of CS, to include studies that document the impact of osilodrostat on the CS treatment paradigm as indicated. Not all included studies reported total scores for HRQoL‐related metrics; some only reported sub‐domain scores. Therefore, our estimated averages of total scores are from a subset of studies and may not represent the overall CS patient population. Additionally, studies that did not report quantitative results for either HRQoL or economic burden were not included. Due to the limited number of studies reporting pre‐ and post‐surgical outcomes, and sex specific differences in HRQoL or economic burden, it is possible that these results do not accurately represent the overall picture. The majority of studies included in this SLR are retrospective, underscoring the need for future prospective studies evaluating the burden of illness in patients with CS over time.

### 4.2 Conclusion

This SLR summarizes the latest evidence on the burden of illness experienced by patients with CS, as well as the associated economic burden of the disease. Notable gaps in the literature were identified. There are few studies measuring the burden of CS specific symptoms in patients and identifying the HRQoL impact of persistent or recurrent disease, and there is a dearth of studies measuring the impact of CS on work productivity and other indirect costs. Further research is needed to understand the holistic burden of CS, particularly as it relates to how symptoms may compound one another. Previous SLRs have reported that HRQoL burden persists despite current treatments, that patients with active or treated CS are worse‐off on certain HRQoL parameters than healthy counterparts, and that there is a need for validated PRO measures to evaluate depression, all of which are in accordance with the findings of this review.^8^, ^50^, ^51^, ^53^ These findings also demonstrate that in CS HRQoL improves after treatment but does not normalize and may require further intervention. Moreover, these findings indicate that additional research is needed to clarify the impact of critical symptoms such as sleep, pain and fatigue on patients' lives. This research also provides a single source for the latest CushingQoL research for a variety of CS etiologies at different time points in the clinical continuum, which may facilitate the real‐world use of this tool in clinical practice. Lastly, this review may encourage further research aimed at developing a more holistic understanding of the burden related to CS.^16^

## AUTHOR CONTRIBUTIONS

**Gabrielle Page‐Wilson:** Conceptualization (equal); supervision (equal); writing – review and editing (equal). **Bhagyashree Oak:** Conceptualization (equal); data curation (equal); formal analysis (equal); methodology (equal); project administration (equal); writing – original draft (equal). **Abigail Silber:** Conceptualization (equal); data curation (equal); formal analysis (equal); methodology (equal); project administration (equal); writing – original draft (equal). **Janetricks C. Okeyo:** Conceptualization (equal); funding acquisition (equal); supervision (equal); writing – review and editing (equal). **Nancy Ortiz:** Conceptualization (equal); funding acquisition (equal); supervision (equal); writing – review and editing (equal). **Mathew O'Hara:** Conceptualization (equal); data curation (equal); formal analysis (equal); methodology (equal); project administration (equal); writing – original draft (equal). **Stephen Moloney:** Conceptualization (equal); funding acquisition (equal); supervision (equal); writing – review and editing (equal). **Eliza B. Geer:** Conceptualization (equal); supervision (equal); writing – review and editing (equal).

## FUNDING INFORMATION

Funding for this study and editorial support was provided by Strongbridge Biopharma, a wholly owned subsidiary of Xeris Biopharma Holdings, Inc. This research was funded in part through the NIH/NCI Cancer Center Support Grant P30 CA008748.

## CONFLICT OF INTEREST STATEMENT

Gabrielle Page‐Wilson, and Eliza B. Geer, were contracted by Strongbridge Biopharma, a wholly owned subsidiary of Xeris Biopharma Holdings, Inc. to provide expert guidance for this study. Bhagyashree Oak, PhD, Abigail Silber and Mathew O'Hara, are employees of Trinity Life Sciences, which was commissioned by Strongbridge Biopharma, a wholly owned subsidiary of Xeris Biopharma Holdings, Inc. to conduct the current study. Janetricks C. Okeyo, CMPP, Nancy Ortiz and Stephen Moloney, MD were employees and shareholders of Strongbridge Biopharma, a wholly owned subsidiary of Xeris Biopharma Holdings, Inc. at the time of this study.

## ETHICS STATEMENT

The data that support the findings of this SLR are available in previously published studies so ethical approval is not applicable.

## Data Availability

The data that support the findings of this SLR are available in the publications included and referenced in this manuscript.

## APPENDIX A

### A.1

**TABLE A1:** List of embase electronic database search terms.

Search No.	Type of term	Search string	Yields as of December 4, 2020
1	Endogenous Cushing's syndrome terms	‘Cushing syndrome’/exp OR ‘cushing disease’/exp OR ‘adrenal cortex adenoma’/exp OR ‘ectopic Cushing syndrome’/exp OR ((‘endogenous’:ab,ti OR ‘ectopic’:ab,ti OR ‘adrenal tumor’:ab,ti OR ‘adenoma’:ab,ti OR ‘pituitary’:ab,ti OR ‘acth‐independent’:ab,ti OR ‘acth‐dependent’:ab,ti OR ‘adrenocortical carcinoma’:ab,ti) AND ‘cushing’:ab,ti AND (‘disease’:ab,ti OR ‘syndrome’:ab,ti))	27,741
2	Economic or humanistic burden terms	‘quality of life’/exp OR ‘economic burden’/exp OR ‘economic evaluation’/exp OR ‘quality of life’:ab,ti OR ‘qol’:ab,ti OR ‘survey’:ab,ti OR ‘questionnaire’:ab,ti OR ‘resource utilization’:ab,ti OR ‘resource use’:ab,ti OR ‘cost’:ab,ti OR economic:ab,ti OR expenditure:ab,ti	2,522,132
3	Final search strategy	#1 AND #2 AND [2015–2020]/py AND [humans]/lim AND [english]/lim AND [abstracts]/lim NOT (([conference abstract]/lim OR [conference paper]/lim OR [conference review]/lim) AND [<1966–2017]/py) NOT ([editorial]/lim OR [erratum]/lim OR [letter]/lim OR [note]/lim OR [animal cell]/lim OR [animal experiment]/lim OR [animal model]/lim OR [animal tissue]/lim OR mouse:ti OR mice:ti OR ‘abstract report’/exp OR ‘book’/exp OR ‘veterinary study’/exp OR ‘genetic model’/exp OR ‘theoretical model’/exp OR ‘molecular model’/exp OR ‘nonbiological model’/exp)	209

**TABLE A2:** List of pubmed electronic database search terms.

Search No.	Type of term	Search string	Yields as of December 4, 2020
1	Endogenous Cushing's syndrome terms	“Pituitary ACTH Hypersecretion”[Mesh] OR “Cushing syndrome”[Mesh] OR “Adrenal Cortex Neoplasms”[Mesh] OR ((“endogenous”[title/abstract] OR “ectopic”[title/abstract] OR “adrenal tumor”[title/abstract] OR “adenoma”[title/abstract] OR “pituitary”[title/abstract] OR “acth‐independent”[title/abstract] OR “acth‐dependent”[title/abstract] OR “adrenocortical carcinoma”[title/abstract]) AND (“cushing”[title/abstract]) AND (“disease”[title/abstract] OR “syndrome”[title/abstract]))	20,781
2	Economic or humanistic burden terms	“Quality of Life”[Mesh]) OR “Cost of Illness”[Mesh] OR “quality of life”[title/abstract] OR “qol”[title/abstract] OR “survey”[title/abstract] OR “questionnaire”[title/abstract] OR “resource utilization”[title/abstract] OR “resource use”[title/abstract] OR “cost”[title/abstract] OR economic[title/abstract] OR expenditure[title/abstract]	1,746,594
3	Final search strategy	#1 AND #2 NOT (“Letter”[Publication Type] OR “Comment”[Publication Type] OR “Editorial”[Publication Type] OR “Published Erratum”[Publication Type] OR “Expression of Concern”[Publication Type] OR “Animal Experimentation”[MeSH Terms] OR “Books”[Mesh] OR “models, genetic”[MeSH Terms] OR “Models, Theoretical”[Mesh] OR “Models, Molecular”[Mesh] OR “genetic techniques”[MeSH Terms])	92

**TABLE A3:** Study eligibility criteria.

Parameter	Inclusion criteria	Exclusion criteria
Population	Patients with endogenous Cushing's syndrome For HRQoL, studies on caregivers of patients with endogenous Cushing's syndrome will also be included	Patients specifically with exogenous Cushing's syndrome Patients with conditions other than endogenous Cushing's syndrome
Intervention and comparator	N/A	N/A
Outcomes	Economic burden of illness Including but not limited to direct and indirect costs of care for patients and caregivers 2 HRQoL Including but not limited to emotional burden, impact on social life, depression, caregiver burden	Only clinical outcomes (no economic or HRQoL burden of endogenous Cushing's syndrome reported)
Study design	Randomized controlled trials Observational studies – prospective, retrospective, longitudinal, or cross‐sectional Economic analyses and models Other published SLRs (for reference checks only – no data extraction will be done for these articles)	Editorials Commentaries Guidelines Genetic studies Case reports/series Animal models Theoretical models Narrative reviews
Geographical limits	N/A	N/A
Database limits	English language Articles with abstracts Human studies	Non‐English language articles Articles without available abstracts
Temporal limits	Original research published from 2015 to 2020 Conference abstracts from 2018 to 2020	Original research and conference abstracts outside temporal limits Records with no abstracts

Abbreviation: HRQoL, health‐related quality of life.

**TABLE A4:** List of PROs included in the studies.

HRQoL type	PROs
General HRQoL	SF questionnaires (SF‐36, SF‐HLQ, SF12v2Score)
	EuroQOL version 5D (EQ‐5D)
	Work role functioning questionnaire (WRFQ)
	Positive and negative affect schedule (PANAS)
Cushing's syndrome specific HRQoL	CushingQOL questionnaire
	Tuebingen Cushing's Disease Quality of Life Inventory (Tuebingen CD‐25)
	QOL‐Cushing's disease (QOL‐CD)
Cognitive, psychological and emotional	Coping Orientation to Problems Experienced Inventory (brief‐COPE)
	Bern Embitterment Inventory (BEI)
	Mental Fatigue Score (MFS‐Score)
	Trail Making Test
	Leiden Brother and Needs Questionnaire (LBNQ‐Pituitary)
	Minnesota Multiphasic Personality Inventory 2 (MMPI‐II)
	Freiburg questionnaire on the use of coping strategies (FKV‐LIS)
Depression and anxiety	Beck Depression Inventory I‐II (BDI‐II)
	Hospital Anxiety and Depression scale D (HADS‐D)
	Montgomery Asberg Depression scale, State Trait Anxiety Inventory (STAI)
	Center For Epidemiological Depression (CES‐D) Questionnaire
Fear and panic	Fear Questionnaire (FQ)
